# Assessment and treatment of headache in primary care: a scoping review

**DOI:** 10.3399/BJGPO.2025.0064

**Published:** 2025-07-30

**Authors:** Jon M Dickson, Aneth Kimaro, Cheong Sxe Chang, Daniel Hind

**Affiliations:** 1 Sheffield Centre for Health and Related Research (SCHARR), School of Medicine and Population Health, The University of Sheffield, Sheffield, UK; 2 School of Medicine and Population Health, The University of Sheffield, Sheffield, UK; 3 Social Care Research and Development, School of Healthcare, University of Leeds, Leeds, UK

**Keywords:** prescribing, neurology, clinical governance, headache

## Abstract

**Background:**

Good quality primary care is essential for the assessment and treatment of headache but there is evidence that primary care for headache is suboptimal.

**Aim:**

To identify the international evidence on the assessment and treatment of headache in adults in primary care.

**Design & setting:**

A scoping review of the published literature following Preferred Reporting Items for Systematic reviews and Meta-Analyses extension for Scoping Reviews (PRISMA-SCR) guidelines, and a narrative review of the evidence.

**Method:**

An electronic search of MEDLINE and Embase (1974–2024) was undertaken. Studies meeting the eligibility criteria were included. Results were grouped by study type and were reported narratively.

**Results:**

In total, 1125 articles were screened, 43 articles underwent full-text review, and 28 articles were included in the final review. Six studies used comparative methods, of which *n* = 3/6 investigated educational interventions. The educational interventions found positive effects on learning, and on outcomes such as diagnosis rates, but the only randomised controlled trial (RCT) did not show any benefits of the intervention. Other comparative studies showed satisfaction with GP with an extended role (GPwER) headache services, benefits from direct access to magnetic resonance imaging (MRI), and benefits from a nurse-led headache service. Twenty-two studies used non-comparative methods, such as surveys and interviews, and investigated approaches to assessment, diagnosis, referral rationale, decision making for prescribing prophylactic medications, educational initiatives, direct access to neuroimaging, GPwER, and nurse-led interventions.

**Conclusion:**

Despite the availability of high quality clinical guidelines on the assessment and management of headache, the evidence shows that its implementation in primary care is problematic and educational interventions are a common focus of published studies. Further research is required to assess the quality of the current evidence and to develop, refine, and deploy interventions that have a signal of efficacy.

## How this fits in

Headache is highly prevalent, and it is a major cause of disability. Primary care has an important role in the assessment and treatment of headache, and by some interpretations of the clinical guidelines, most cases of headache should be exclusively assessed and managed in primary care and referral to a specialist should be rare. To deliver good patient outcomes, the primary care workforce needs sufficient skills and capacity, but the evidence shows that many people with headaches receive suboptimal primary care with poor symptom control, under-use of key treatments, such as migraine prophylaxis and triptans, and high rates of medication overuse headache. There have not previously been any attempts to review the evidence on primary care headache management for effective interventions nor to identify areas that require more research. This scoping review addresses that knowledge gap.

## Introduction

Headache affects around 47% of people globally^1^ and it is among the top 10 causes of disability, according to the World Health Organization (WHO).^2^ In the UK, migraine, which is the most common type of headache, affects 10 million people, that is, one in seven adults. Primary care is the first point of contact for people seeking medical care for headaches, accounting for one in 10 consultations.^3^ By some interpretations of clinical guidelines, most cases of headache should be managed exclusively in primary care and referral to a specialist should be rare. Waiting times for specialist clinics increased from 15–29 weeks between 2021 and 2023 in the NHS and are likely to be even higher now. Only 62% of integrated care systems (ICS) in England have a specialist headache clinic.^4^

Despite the importance of headaches to patients and to the health service, a 2014 report highlighted insufficient education and training resources for non-specialists in the NHS.^5^ Primary care clinicians sometimes struggle with diagnosis and treatment leading to suboptimal outcomes^6–9^ and they may benefit from extra education and training, new guidelines, and tools^10,11^ to improve the care they deliver, to improve the quality of specialist referrals,^12^ and to reduce unnecessary referrals.^13^

A recent review of educational initiatives highlighted the need for innovative, evidence-based methods for content delivery, knowledge assessment, and evaluation,^14^ with the aim of enhanced patient outcomes, and improved cost-effectiveness.^15–17^ Several studies and reports have explored optimal care pathways^18^ and innovations such as providing GPs with direct access to magnetic resonance imaging (MRI) scans.^4,19^ However, there have not been any attempts to review the evidence pertaining to primary care for people with headaches, to explore which topics are important for clinicians, to explore interventions and their effectiveness, and to identify areas that require more research. A scoping review is the ideal method to identify the extent and nature of a body of evidence, to identify gaps, and to guide future research and ultimately to improve patient care. Therefore, we set ourselves the aims of undertaking a scoping review of the published literature and producing a narrative review of the evidence that we found.

We looked for international evidence to ensure that we captured the best possible evidence from across the world, despite the potential for limited applicability between some countries. And we chose to focus on adults, excluding studies on children because there are significant differences in the differential diagnosis in the two groups; access to neuroimaging for children is usually restricted to specialists, and the threshold for referral is lower in children.

## Method

This review was conducted in line with the Joanna Briggs Institute (JBI) methodology for scoping reviews and is reported according to the Preferred Reporting Items for Systematic Reviews and Meta-Analysis extension for Scoping Reviews (PRISMA-SCR) statement.^20^ The protocol was set before conducting the review; it was not registered or published.

### Eligibility criteria

The review was structured using the Population–Context–Concept (PCC) framework;^21^ see Table 1 for details.

**Table 1.** Inclusion and exclusion criteria

	Inclusion criteria	Exclusion criteria
**Population**	Adults seeking primary medical care for headaches. Care delivered by GPs or primary care nurses.	Children seeking primary medical care for headaches. Care delivered by other primary healthcare professionals. Studies not focused on headaches, but focused on diseases such as brain tumours or giant cell arteritis, which can cause headache, but where diagnosis or management of the headache was not the focus of the article.
**Context**	Primary care settings (some studies conducted in secondary care were included if they focused on the primary care perspective, for example, studies evaluating GPs’ reasons for referrals).	Studies conducted outside primary care settings, such as hospitals or hospital-run clinics, were usually excluded.
**Concept**	Focus on headaches in terms of assessment and management in primary care. This included practitioners’ knowledge and attitudes, variations in practice, referral reasons or thresholds, the rationale for treatment choices, training opportunities and learning needs, GPs with an extended role (GPwER), health economics, capacity, and the role of neuroimaging.	Studies based solely on incidence and prevalence of headache in primary care. Studies focused on patient perspectives were also excluded.
**Type of studies**	Primary research such as randomised controlled trials (RCTs), cohort studies, qualitative studies, surveys, audits, and service evaluations. Peer-reviewed articles and conference abstracts. Articles were included without limitations on publication year or country.	We excluded editorials, opinion pieces, discussion articles, tutorials, case studies, review articles, and guidelines. Non-English language articles were excluded.

### Information sources, search strategy, and article selection

We searched MEDLINE and Embase from 1974–24 May 2024. The full MEDLINE and Embase search strategies are outlined in Appendix S1.

The search results were uploaded to Rayyan^22^ and duplicates removed. Two reviewers screened the title and abstract for eligibility, retrieving full-text articles when necessary. In instances where the title and abstract were ambiguous, full-text articles were retrieved. Records were included if they met the inclusion criteria and none of the exclusion criteria as agreed on by two reviewers (AK and WCSC). Conflicts were resolved by a third reviewer (DH) through discussions or meetings. We did not critically appraise study quality but used study design as a proxy for evidential quality.

### Data extraction, data items, and narrative review

A standardised data extraction form was developed. Two reviewers (AK and WCSC) worked independently to extract study details, and an additional reviewer (DH) resolved any conflicts. For all studies, we extracted data on the country of origin, setting, publication type, study design, and type of headache treated. For comparative studies discussing interventions, we extracted information on the intervention and comparisons used, tools for measuring outcomes, and findings. For non-comparative studies, we gathered information on the findings, themes, and the authors’ recommendations.

The results are presented in traditional narrative form.^23,24^ We did not undertake a formal narrative synthesis, instead we aimed to summarise the studies as a body of evidence while preserving their idiosyncratic and unique nature. This allowed us to accommodate the different research questions, designs, and contexts of individual studies, which are presented in tabular summaries.

## Results

### Selection of sources of evidence

**Figure 1.**
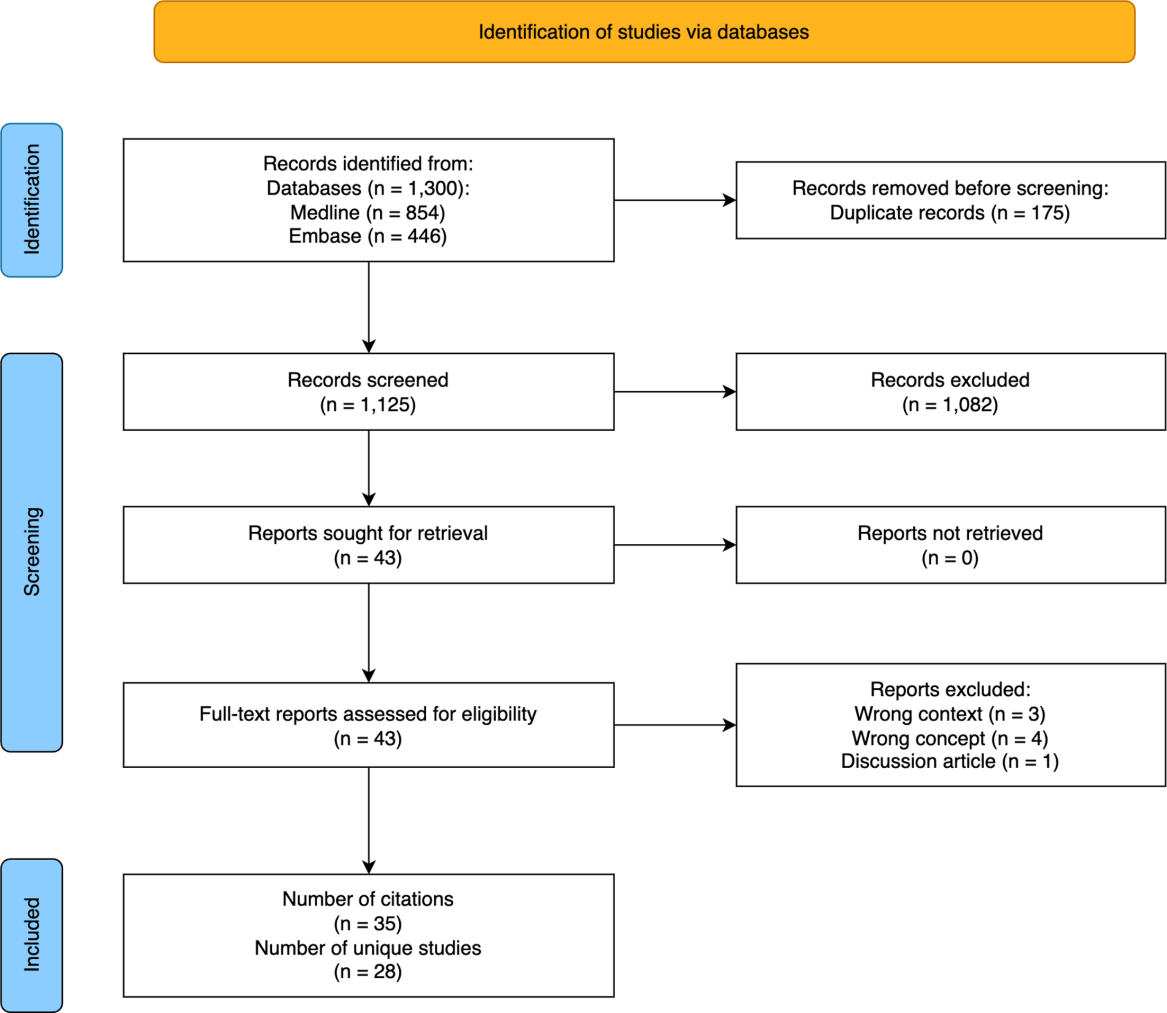
Preferred Reporting Items for Systematic Reviews and Meta-Analyses (PRISMA) flowchart

Initial database searches identified 1125 records after the removal of duplicates (see Figure 1). Forty-three articles fulfilled the criteria using the title and abstract. The full text of these articles was retrieved and assessed. Eight articles were excluded at this stage for focusing on the following: secondary care perspective of headache referrals (*n* = 3); the prevalence of headache in primary care (*n* = 1); patients’ perspectives (*n* = 3); and not being primary research (*n* = 1). This left 35 articles, of which seven articles were reporting similar results to another already included study and so were excluded.^25–31^ In total, 28 unique studies were included in the final review.

### Characteristics of sources of evidence

Twenty-five studies were conducted in Europe (*n* = 25),^3,4,8,32–53^ two studies in Africa (*n* = 2),^54,55^ and one study in Australia (*n* = 1).^56^ Among European countries, 10 studies were conducted in the UK (*n* = 10),^3,4,33,43–46,48,51,52^ three studies each in The Netherlands (*n* = 3)^8,34,35^ and Norway (*n* = 3),^40,50,53^ two studies were conducted across multiple European countries (*n* = 2),^39,42^ and one study each in Germany (*n* = 1),^49^ Italy (*n* = 1),^36^ Denmark (*n* = 1),^47^ Switzerland (*n* = 1),^38^ Spain (*n* = 1),^41^ Russia (*n* = 1),^37^ and Estonia (*n* = 1).^32^

We classified the studies methodologically as follows: comparative studies (*n* = 6)^4,32–35,53^ and non-comparative studies (*n* = 22).^3,8,36–52,54–56^ Among the non-comparative studies, 17 used quantitative methods (*n* = 17)^3,36–48,54–56^ and five used qualitative methods (*n* = 5).^8,49–52^

Twenty-three studies included in this review were publications in peer-reviewed journals and five were conference proceedings (*n* = 5).^37,41,46,48,55^

The majority of studies investigated patients with headaches of all causes and a minority investigated specific headache types; for example, migraine, or tension-type headache. In this article we use the phrase ’*headache (all causes*)’ to denote the former group.

See Table 2 for the full list of study characteristics.

**Table 2.** Summary of study characteristics

First author, publication year	Country of origin	Setting	Publication type	Study design	Population
Bianco, 2005^36^	Italy	General practice	Journal article	Survey	Migraine
Bösner, 2014^49^	Germany	General practice	Journal article	Qualitative interviews	Headache (all causes)
Braschinsky, 2016^32^	Estonia	General practice	Journal article	Non-randomised intervention	Headache (all causes)
Carlsen, 2024^47^	Denmark	General practice	Journal article	Audit	Headache (all causes)
Dabilgou, 2021^54^	Burkina Faso	District hospitals	Journal article	Survey	Migraine
Dekker, 2012^8^	The Netherlands	General practice	Journal article	Qualitative interviews	Migraine
Elliot, 2011^45^	UK	GPwSI	Journal article	Retrospective data extraction^a^	Headache (all causes)
Elsherif, 2022^46^	UK	OPN	Conference presentation	Retrospective data extraction^a^	Headache (all causes)
Fokin, 2011^37^	Russia	OPC	Conference presentation	Survey	Headache (all causes)
Frich, 2014^50^	Norway	General practice	Journal article	Qualitative interviews	MOH
Gantenbein, 2013^38^	Switzerland	PCP	Journal article	Survey	Headache (all causes)
Klippel, 2008^39^	Multiple countries^b^	General practice	Journal article	Survey	Migraine
Kristoffersen, 2021^40^	Norway	General practice	Journal article	Survey	Headache (all causes)
Latinovic, 2006^3^	UK	General practice	Journal article	Retrospective data extraction^a^	Headache (all causes)
Lip, 2013^48^	UK	General practice	Conference presentation	Audit	Headache (all causes)
Morgan, 2007^51^	UK	General practice	Journal article	Qualitative interviews	Headache (all causes)
Pascual, 2009^41^	Spain	General practice	Conference presentation	Survey	Migraine
Ridsdale, 2008^33^	UK	General practice and NC	Journal article	Non-randomised intervention	Headache (all causes)
Ryvlin, 2021^42^	Multiple countries^c^	General practice	Journal article	Survey	Chronic migraine
Sanai, 2022^55^	Tunisia	General practice and FM	Conference presentation	Survey	Headache (all causes)
Schjøtt, 2024^53^	Norway	General practice	Journal article	Non-randomised intervention	Migraine
Simpson, 2010^43^	UK	PCP	Journal article	Survey	Chronic headache
Smelt, 2012^26^	The Netherlands	General practice	Journal article	Randomised trial	Migraine
Sun, 2013^56^	Australia	General practice	Journal article	Survey	Headache (all causes)
Taylor, 2012^4^	UK	General practice and NC	Journal article	Retrospective cohort comparison	Headache (all causes)
Thomas, 2010^44^	UK	General practice	Journal article	Survey	Headache (all causes)
Underwood, 2017^52^	UK	PCP	Journal article	Qualitative interviews	Headache (all causes)
Veenstra, 2016^35^	The Netherlands	General practice	Journal article	Non-randomised intervention	Migraine

^a^Retrospective data extraction from database or referral letters. ^b^Germany, Portugal, and Belgium. ^c^France, Germany, Italy, Spain, and the UK. FM = family medicine. GPwSI = GP with special interest. MOH = medication overuse headache. NC = neurology clinic. OPC = outpatient clinic. OPN = outpatient neurology. PCP = primary care practice.

### Comparative studies

There were six comparative studies that are summarised in Table 3.

**Table 3.** Comparative studies included in the review with intervention, outcomes, and results

Study ID, first author	Population	Study design	Intervention and comparison		**Outcomes**	**Results**
			Educational interventions	Non-educational intervention		
**Smelt, 2012^26^**	Migraine	Randomised controlled trial	I: GPs received headache training and additional learning materials. C: Usual care by GP		Headache Impact Test (HIT-6). Migraine characteristics (frequency, severity, and duration of migraine attacks; absence from work; and medication use).	At 6 months, HIT-6 scores were similar between groups, but by 12 months, the intervention group reported a greater decrease. No significant differences in attack characteristics, headache days, or work absences between the intervention and control groups. GP training for migraine management was not cost-effective compared with usual GP care.
**Ridsdale, 2008^33^**	Headache (all causes)	Observational study		I: GPwSI service. C: Hospital neurologist	HIT-6. Patient satisfaction. Cost-effectiveness.	No significant difference in HIT-6 scores between patients referred to a neurologist and to GPwSI service. Patients were more satisfied with the GPwSI service. GPwSI consultation costs were lower than those for a neurologist.
**Schjøtt, 2024^53^**	Migraine	Non-randomised controlled trial	I: Virtual continuing medical education (CME) on rational treatment of migraine. C: In-person CME		Self-perceived learning outcomes.	No significant difference in perceived increase in knowledge between virtual and in-person CME attendees before and after, although virtual attendees tended to have a higher proportion of positive perceptions. CME attendees, 80–88% of GPs, reported positive self-perceived learning outcomes from both in-person and virtual sessions.
**Taylor, 2012^4^**	Headache (all causes)	Retrospective cohort study		I: GP direct access to magnetic resonance imaging (MRI). C: MRI requested from neurology clinics	Radiological findings.	Patient satisfaction was high, and there was a cost reduction in the direct access pathway group. No significant differences in major abnormalities, incidental findings, or ischaemic lesions were found between the two cohorts.
**Braschinsky, 2016^32^**	Headache (all causes)	Non-randomised controlled trial	I: 2-day educational course with supporting material. C: Patients treated before the intervention	.	Referral rate. GPs diagnosis, treatment and diagnostic tests. Patient satisfaction and wellbeing assessment.	More diagnoses of types of headache for example, migraine versus tension-type headache. Fewer tests were ordered and there was an increase in initiation of treatment. No significant change in patients’ satisfaction before and after intervention. No significant reduction in referrals.
**Veenstra, 2016^35^**	Migraine	Non-randomised controlled trial		I: Management by a nurse under GP supervision. C: Management by GP	Referral rate to the hospital. Changes in HIT-6 score. Changes in mean monthly headache days. Changes in patients’ satisfaction compared with baseline.	Fewer patients with migraine in the intervention group were referred to a neurologist. No significant change in HIT-6 score between groups. Patients in the intervention group reported a significant decrease in monthly headache days. No significant difference in patient satisfaction scores between groups, but the intervention group showed a trend towards higher satisfaction.

**Explanation**: I = Intervention. C = comparators.

Three of the studies investigated patients with migraine, and three investigated headaches of all causes. Four studies were trials (randomised controlled trial = 1; non-randomised controlled trials = 3), one was an observational study, and one was a retrospective cohort study. Three studies looked at educational topics, and three studies looked at non-educational topics.

Most of the educational studies reported positively on their effects. Schjott *et al* ^53^ reported positive self-perceived learning from a medical educational on treatment of migraine. Braschinsky *et al* ^32^ reported higher diagnosis rates, reduced investigations, and more initiation of treatment from a 2-day educational course, but the study did not show improvements in patient satisfaction, or reduction in referrals. Smelt *et al*,^34^ the only randomised controlled trial that we found, did not show any benefit of an educational intervention, and concluded that psychological distress among the study population was an important confounder.

Ridsdale *et al* ^33^ showed that patients were more satisfied with a GP with a special interest (GPwSI) service than a hospital neurologist service, and that the costs of the GPwSI service were lower. Taylor *et al* ^4^ showed that direct access to MRI for GPs led to high patient satisfaction, cost reductions, and no difference in the findings of the scans between the groups. Veenstra *et al* ^35^ showed reduction in referrals and reduced headaches for the nurse-led intervention compared with management by a GP, but no significant difference in patient satisfaction.

### Non-comparative studies

#### Quantitative

There were 17 non-comparative studies that utilised quantitative methods. The study population, study design, and the focus of each study is summarised in Table 4.

**Table 4.** Summary of non-comparative quantitative studies

Study ID, first author	Population	Study design	Assessment strategies			Treatments		Referral	Education	
			Imaging	Headache diary	Guidelines	Acute treatment	Prophylactic treatment	Pattern and destination	Training needs	Training available
Bianco, 2005^36^	Migraine	Surveys				x				x
Dabilgou, 2021^54^	Migraine	Surveys	x	x	x	x	x	x		
Pascual, 2009^41^	Migraine	Surveys				x	x		x	
Ryvlin, 2021^42^	Chronic migraine	Surveys	x	x	x	x	x	x		
Klippel, 2008^39^	Migraine	Surveys				x		x		
Gantenbein, 2013^38^	Headache (all causes)	Surveys				x		x	x	
Fokin, 2011^37^	Headache (all causes)	Surveys							x	
Kristoffersen, 2021^40^	Headache (all causes)	Surveys	x	x	x	x	x	x		
Sanai, 2022^55^	Headache (all causes)	Surveys				x		x	x	
Simpson, 2010^43^	Headache (all causes)	Surveys	x					x		
Sun, 2013^56^	Headache (all causes)	Surveys	x					x		
Thomas, 2010^44^	Headache (all causes)	Surveys	x					x		
Elliot, 2011^45^	Headache (all causes)	Retrospective cohort study	x							
Elsherif, 2022^46^	Headache (all causes)	Retrospective cohort study						x		
Latinovic, 2006^3^	Headache (all causes)	Retrospective cohort study				x		x		
Carlsen, 2024^47^	Headache (all causes)	Audits		x		x	x	x		
Lip, 2013^48^	Headache (all causes)	Audits				x		x		

Eight studies investigated assessment strategies used by GPs (*n* = 8).^40,42–45,47,54,56^ Seven of these studies looked at assessment strategies involving the use of imaging (*n* = 7).^40,42–45,54,56^ and four explored the use of patient headache diaries (*n* = 4).^40,42,47,54^ Three studies reported the use of guidelines and recommendations.^40,42,54^

Eleven studies explored GPs’ behaviour and choices in prescribing acute treatments (*n* = 11)^3,36,38–42,47,48,54,55^ and five studies examined prophylactic treatments (*n* = 5).^40–42,47,54^

Fourteen studies investigated GP referrals (*n* = 14).^3,4,38–40,42–44,46–48,54–56^ Of these, five studies reported that GPs referred patients to specialists (*n* = 5),^39,40,42,48,54^ four studies involved referrals to both specialists and imaging services (computed tomography [CT] and MRI scans) (*n* = 4),^43,44,47,56^ two studies referred patients to neurology clinics (*n* = 2),^3,46^ and one study involved referrals to imaging services only (*n* = 1).^38^ The most common reasons for these referrals were better treatment options for patients (*n* = 6),^39,40,42,48,54,56^ diagnosis or diagnosis confirmation (*n* = 3),^42,54,56^ diagnostic uncertainty (*n* = 2),^46,57^ and seeking expert advice (*n* = 1).^38^

Five studies investigated training and education for GPs. Of these, one study reported on available continuing medical education (CME) for GPs (*n* = 1)^36^ and four studies focused on continuing training or learning needs (*n* = 4).^37,38,41,55^ Two studies found a significant proportion of GPs desired additional education on headache management through practice-oriented workshops (*n* = 1)^38^ and postgraduate courses (*n* = 1).^55^ One study highlighted common mistakes in headache evaluation and management by GPs, emphasising the need for enhanced training (*n* = 1).^41^ Another study indicated that training could lead to a 15–20% increase in headache diagnosis and management efficiency (*n* = 1).^37^ One study highlighted the lack of GP awareness of evidence-based medicine (EBM) owing to difficulties in interpreting and accessing relevant information (*n*= 1).^36^ Another study explored cost-effectiveness, noting that GPs with direct access to CT scans were cost-saving by reducing unnecessary referrals to secondary care (*n* = 1).^43^

#### Qualitative

There were five non-comparative studies that utilised qualitative interviews; these are summarised in Table 5.

**Table 5.** Non-comparative qualitative studies Included with themes explored and results

Study ID, first author	Population	Aims	Results	Authors’ recommendation
**Frich, 2014^50^**	Medication overuse headache	Explore GPs’ experiences, feasibility, and efficacy of using brief interventions (BIs) in the management of medication- overuse headache (MOH).	GPs faced challenges in helping patients understand MOH but using it as a formal diagnosis helped change patients’ perceptions. The BI strategy is feasible and effective in changing patients’ perceptions and medication habits, but its success depends on the GP–patient relationship. To ensure a successful intervention, GPs must address patients’ emotions, counter misconceptions about over-the-counter medications, and use reliable visual aids to enhance patient understanding.	Outside a study situation, a GP’s alliance with a patient over time may be an important additional factor for success of BI. However, this requires further studies, and a prerequisite is that the GP is aware of the patient’s risk of MOH in advance.
**Dekker, 2012^8^**	Migraine	Investigate GPs’ decision-making processes regarding prophylactic migraine medication.	GPs underuse prophylactic migraine medication owing to concerns about side effects, effectiveness, and patient factors. Prophylactic prescriptions are often based on patient preferences and GP experience, rather than national guidelines, causing delays.	These factors should be addressed in guideline setting and postgraduate education. Finally, some aspects of the findings of this study need further exploration, and some deserve quantification.
**Bösner, 2014^49^**	Headache (all causes)	Explore how GPs diagnose and manage headaches in primary care.	GPs often rely on long-term patient relationships, intuition, personal experience, and first impressions when diagnosing headaches. For self-limiting headaches without red-flag symptoms, GPs may monitor the patient’s condition over time and use therapeutic trials to confirm if the headache is benign, reducing the need for extensive diagnostic testing.	This study’s findings underline the need for further guidance in the workup of patients with headache. This may be in the form of effective strategies for handling uncertainty including guidance on specialist referral or the development of simple guidelines that allow making an exact diagnosis in the specific context of primary care.
**Morgan, 2007^51^**	Headache (all causes)	Explore GPs’ decisions to refer patients with headache to specialists.	GPs’ decisions to refer patients with headache to specialists are influenced by patient anxiety, pressure, clinical experience, confidence, and the availability of local services, including access to GPs with specialist interest or charity-funded clinics. GPs with more resources tended to refer patients to these alternatives rather than specialists. Some GPs believed patients have a *‘right to referral*,’ to address patients’ anxiety while others saw it as a means of providing reassurance.	Reducing specialist neurological referrals requires further training and support for some GPs in the diagnosis and management of headache. To reduce clinical uncertainty, good clinical prediction rules for headache and alternative referral pathways are required.
**Underwood, 2017^52^**	Headache (all causes)	Explore GPs’ views in diagnosing headaches, specifically their use of direct-access magnetic resonance imaging (MRI) scans Explore the outcome of GPs managing and diagnosing patients with headaches after an educational session by a GP with a special interest (GPwSI).	Reassurance is a key factor in deciding patient referral for scans, but it doesn’t always alleviate anxiety in patients with significant symptoms and psychological issues. Normal scans help in effective headache management. GPs face challenges interpreting radiology reports, especially with incidental findings. Post-education with GPwSI, GPs reported improved confidence in patient management.	An educational component rolled out alongside direct-access scanning, emphasising a holistic approach that empowers and reassures patients, may be as important as more traditional teaching around diagnosis and medication.

From the three studies on managing all headache types, several themes emerged. One study (*n* = 1)^50^ highlighted GPs’ views on the diagnostic approach, including their understanding of patients and their medical history, reliance on intuition, personal experience, and the passage of time.^49^ One study (*n* = 1)^51^ reported disparities in GPs’ confidence in patient referrals and the diversity of referral approaches, considering factors such as identifying life-threatening conditions, tolerance for uncertainty, beliefs about patient entitlement to referrals, perception of referral benefits, availability of local services, including GPwSI in clinics funded by charities. GPs were often compelled to make referrals owing to patient anxiety.^51^ Another study (*n* = 1)^52^ found that GPs used scans to guide management, address uncertainty, and facilitate preventive treatment discussions, even without a perceived benefit in reassuring patients. GPs who received prior teaching and education were more confident in managing patients and interpreting radiology reports compared with those who received no additional education.^52^

One study (*n* = 1)^8^ focusing on the management of migraine highlighted GPs’ decision-making processes in administering prophylactic medication when acute medication provides insufficient relief.

Another study (*n* = 1)^50^ discussing medication overuse headache reported the importance of considering patient autonomy, the benefits of reducing patient resistance to medication-induced headaches by formally diagnosing it as ’medication overuse headache’. It also highlighted the significance of building a strong alliance with patients to effectively integrate brief interventions (BIs) into regular consultations for self-management of headaches by constantly reshaping patients’ perceptions of their headaches and medication use.

## Discussion

### Summary

We identified 28 studies that met our criteria. Six studies used comparative methods, three of which investigated educational interventions. The educational interventions showed positive effects on learning and patient outcomes, such as diagnosis rates, but the only RCT found no significant benefits. Other comparative studies highlighted satisfaction with GP with an extended role (GPwER) headache services, benefits from direct MRI access, and advantages of nurse-led headache services. Twenty-two studies used non-comparative methods, such as surveys and interviews, exploring assessment and/or diagnosis, referral rationale, decision making for prescribing prophylactic medications, educational initiatives, direct neuroimaging access, and GPwSI and nurse-led interventions.

Despite high quality clinical guidelines for headache assessment and management, implementation in primary care is problematic, with educational interventions often being the focus of studies. There is evidence to indicate that an educational intervention delivered in primary care could improve patient outcomes, improve confidence among GPs, reduce unnecessary investigations, reduce referrals to secondary care, and reduce costs. Further research is needed to assess the quality of current evidence and refine interventions with a signal of efficacy, and to design definitive trials.

### Strengths and limitations

As far as we are aware this is the first review in the published literature on the assessment and management of headaches in primary care. It was conducted according to gold standard methods (PRISMA-SCR and JBI) ensuring a transparent, systematic, credible, and replicable approach.^20,58^ We comprehensively identified the available literature, providing an overview of each article and a narrative review of the evidence, and we have identified knowledge gaps and made suggestions for further research.

Scoping reviews often identify methodologically heterogenous literature, which makes comprehensive and coherent quality assessment across the different methods challenging. Our study was not externally funded, limiting the capacity of the review team. We did not critically appraise study quality, but we reported the design of each study as a proxy of evidential quality. Our review was limited to English-language studies, potentially omitting valuable research in other languages and introducing language bias, resulting in an incomplete reflection of the full body of international evidence. Owing to capacity constraints, our search was confined to two databases meaning that we may have missed articles that were indexed in other databases. Including additional databases, such as the Cumulative Index to Nursing and Allied Health Literature (CINAHL), and sources of grey literature could have provided more comprehensive coverage.

### Comparison with existing literature

Although there are many good quality clinical guidelines for headache, most of these do not specify which sector (primary care, secondary care, tertiary care) should provide the elements of care that are recommended. People affected by headache are often living with multimorbidity (physical and psychological) and polypharmacy that requires a generalist whole-person approach, which makes primary care the optimal sector to deliver most care for people with headache. Despite this, the literature is dominated by secondary and tertiary care perspectives, which is not useful in a primary care context, and which creates an epistemic bias. This review redresses that bias and presents the evidence that is relevant to primary care.

### Implications for research and practice

The articles in this review provide evidence for GPs, clinicians, commissioners, managers, and policymakers. While we did not formally assess evidence quality, we identified studies, particularly those using comparative methods with outcome data, suggesting that educational interventions in primary care can improve patient outcomes, boost GP confidence, and reduce unnecessary investigations, referrals, and costs. A key implication of this review is the need for formal quality assessment, further research, and the development of effective interventions.

The best design of service reconfigurations or interventions based on the evidence available is open to interpretation. Many of the problems with delivery of care for people with headaches reflect lack of capacity across the whole system and not specific problems with primary care. Many issues with headache care delivery stem from system-wide capacity limitations, not specific problems in primary care. New services must involve whole-system modelling, including health economics, to ensure any additional costs are justified by savings in areas such as emergency care, referrals, and neuroimaging. Clear boundaries must be established between primary and secondary care to prevent the current unproductive disputes that currently prevail. Once boundaries are established, structures should be put in place to encourage strong relationships with specialists who can provide advice, support, and specialist review when necessary.

## Data

Data is not available.
